# Do gay and bisexual men who conceal their same-sex behavior prefer different kinds of health services? Findings across four cities to inform client-centered HIV prevention in China

**DOI:** 10.1186/s12889-019-7990-8

**Published:** 2020-01-06

**Authors:** Rong Fu, Bryan A. Kutner, Yumeng Wu, Lu Xie, Siyan Meng, Jianhua Hou, Yuzhou Gu, Huifang Xu, Huang Zheng, Na He, Kathrine Meyers

**Affiliations:** 1 Guangzhou Center for Disease Control and Prevention, Guangzhou, China; 2 HIV Center for Clinical and Behavioral Studies, New York State Psychiatric Institute and Columbia University, New York, USA; 3 Aaron Diamond AIDS Research Center, The Rockefeller University, 455 1st Avenue, 7th Floor, New York, NY 10016 USA; 4 Institution of HIV/AIDS, The First Hospital of Changsha, Changsha, China; 5 School of Public Health, Fudan University, Shanghai, China; 6 Beijing Youan Hospital, Capital Medical University, Beijing, China; 7 Shanghai CSW & MSM Center, Shanghai, China

**Keywords:** Sexual health service, Healthcare providers (HCPs), Men who have sex with men (MSM), HIV, Sexually transmitted infections (STIs)

## Abstract

**Background:**

In China, addressing disparities in the HIV epidemic among men who have sex with men (MSM) requires targeted efforts to increase their engagement and retention in prevention. In an effort to advance MSM-friendly HIV services within China, and informed by community-based partnerships, we tested whether MSM who have ever versus never disclosed their same-sex behavior to healthcare providers (HCP) differ in sociodemographic and behavioral characteristics as well as the qualities of sexual health services each group would prefer to access.

**Methods:**

We conducted a cross-sectional survey among HIV-negative MSM who went to MSM-focused voluntary counseling and testing clinics in four cities in China. The survey was anonymous and collected information on sociodemographic characteristics, testing behaviors, sexual-health related behavior, and sexual health service model preferences.

**Results:**

Of 357 respondents, 68.1% participants had ever disclosed same-sex behavior to HCPs when seeking advice for sexual health. Younger age (*aOR* = 1.04; *95% CI*: 1.01-1.08), and worry of HIV acquisition (*aOR* = 1.39; *95% CI*: 1.05–1.84) were associated with higher odds of past disclosure. The availability of comprehensive sexual health services was one of the most valued characteristics of the ideal sexual health clinic. Those who ever disclosed and never disclosed differed significantly in their ranking of the importance of three out of ten dimensions: sexual health counseling services available (*M* = 3.99 vs. *M* = 3.65, *p* = .002), gay identity support available (*M* = 3.91 vs. *M* = 3.62, *p* = .016) and clinic collaborates with a gay CBO (*M* = 3.81 vs. *M* = 3.56, *p* = .036).

**Conclusions:**

Our hypothesis that MSM who had disclosed versus never disclosed same-sex behavior would differ in the value they placed on different dimensions of sexual health service was partially borne out. As health authorities in China decide on implementation models for pre-exposure prophylaxis (PrEP) delivery and specifically within which institutions to integrate PrEP services, the preferences of target populations should be considered to develop comprehensive, patient-centric and LGBT-friendly services.

## Background

In many areas of the world where homosexuality remains illegal or stigmatized in structural and social ways, gay, bisexual and other men who have sex with men (MSM) largely decline to disclose their sexuality and same-sex behavior to healthcare providers (HCPs) [1]. This concealment protects MSM against the deleterious effects of experiencing further maltreatment [2], but also means that HCPs miss opportunities to tailor sexual health services to intervene on the disproportionate burden of STIs and HIV experienced by MSM as a key population [3]. While environmental factors in healthcare settings, such as HCPs’ negative attitudes and verbal harassment of MSM, may limit men’s willingness to disclose same-sex behavior [2], at least some of these factors are amenable to change [3]. For example, broadening the focus from HIV to sexual health and including mental health services related to sexuality might lead to improved engagement of MSM across the HIV continuum [4].

In China, over the course of the rapid expansion of a nationwide epidemic of HIV and STIs during the past two decades, MSM are now recognized as a disproportionately affected group [5, 6]. In the past ten years, Chinese funding bodies have scaled up HIV testing services for MSM in both healthcare settings and CBOs [5]. However, delayed and suboptimal HIV and syphilis testing and ART initiation remain common [7–9]. A very high proportion of Chinese MSM also experience stigma in healthcare settings and do not disclose sexual orientation to their health professionals [10, 11]. Reorienting existing health services to affirm MSM sexuality, in line with the World Health Organization (WHO) definition of sexual health [12] and recommendations for sexual health service models specific to MSM [3, 13] may lessen the disproportionate burden of HIV and STIs by improving engagement and retention in services [14].

Previous studies have evaluated sexual health services by focusing on HCPs’ perception of MSM [13] and MSM disclosure in the context of healthcare discrimination [11]. While most Chinese MSM prefer to go to MSM-focused CBOs for HIV counselling and testing [15, 16], little is known about how these men seek HIV testing services in this high stigma setting or perceive sexual health services. In this study, we aimed to assess two aspects of sexual health services: A) demographic and behavioral characteristics associated with disclosure of same-sex behavior to HCPs, and B) whether disclosure was associated with different priorities for sexual health services among MSM.

## Methods

### Study design and sampling methods

Our study team held a series of stakeholder meetings in China with gay community leaders, HIV prevention intervention staff at municipal Centers for Disease Control and Prevention (CDC), and antiretroviral and STI clinic providers to discuss potential models for sexual health services for Chinese MSM. Importantly, stakeholders posited that MSM who conceal their same-sex behavior may differ in the qualities they prioritize within sexual health services that they would like to access, compared to counterparts who have already disclosed their same-sex behavior within a health care setting.

To test this hypothesis, between February and May 2018, we conducted a cross-sectional study in four cities (Shanghai, Beijing, Changsha, and Guangzhou) in a convenience sample of Chinese MSM. Staff at gay-oriented CBOs recruited participants through four methods: voluntary counselling and testing (VCT) clinics with posters, peer networks, outreach at gay venues with fliers, and online through CBOs’ WeChat posts. Four trained research coordinators (RF, LX, JH, and SM) assessed eligibility criteria and screened men who identified as: 18 years or older, assigned male at birth, had ever engaged in sex with another man, and HIV-negative by self-report.

Computer-based surveys were administered in person by either CBO staff or research coordinators or self-administered by participants themselves. Participants gave written informed consent prior to survey administration and received compensation of $7.40 USD or equivalent condom and lubricant as compensation for their time. Recruitment halted after reaching the pre-specified sample of 700, of whom 362 were recruited through VCT clinic. We restricted the sample to VCT-recruited participants for this research question. Protocol and all study procedures were approved by the ethics review committee of Fudan University (Shanghai, China).

### Measures

The survey was anonymous and collected information on sociodemographic characteristics and sexual health related behavior, including testing behaviors, sexual risk and risk perception, and preferences for dimensions of a sexual health service model. Most survey items were adapted from an existing assessment instrument for Chinese MSM [17].

The outcome measure was lifetime disclosure of same-sex behavior to HCPs when seeking advice for sexual health issues. This advice-seeking could relate to bleeding, pain, itching, infections in genital or anal areas, or sexual dysfunction.

Sociodemographic information included *age*, *city currently living in*, *residence* (local or non-local), *education*, *employment status*, *student status*, *household composition*, *marital status*, and *average monthly income* (low and middle income, middle income, and high income) [18].

We collected detailed information on sexual health related behavior. Participants self-reported if they *ever had a sexual health issue that interfered with sex* (yes or no) as well as *ever experienced sexual health issues and went to see HCPs* (yes or no). We broadly defined HCPs based on classification from WHO [19] and MSM-specific guidelines [4], as either health professionals, or lay HIV counselors. The survey also asked participants about their *frequency of HIV and STI testing* and *sexual risk behavior*. Variables associated with sexual risk behavior within the past 6 months included *number of male sex partners*, *HIV-positive male partner*, *condomless anal sex*, *commercial sex behavior with male sex partners*, *STI symptoms/diagnosis*, *recreational drug use*, *sexual positioning*, and *group sex with male sex partners*. We assessed *self-perception of HIV risk* with the following questions: “In five years, how likely do you think you are to become infected with HIV” (ranging from *1 very unlikely* to *4 very likely*); “In the past two years, have any of your friends or people you know become infected with HIV” (no, yes, I don’t know); and “How worried are you of becoming infected with HIV” (ranging from *1 not worried at all* to *4 very worried*).

After iterative discussions with GPP stakeholders in four participating cities, we developed an MSM-specific sexual health service model that participants could evaluate by prioritizing the model’s hypothetical “ideal” dimensions. Participants ranked the relative importance of each dimension of this clinic-based sexual health service model using a 5-point Likert scale (ranging from *1 not at all important* to *5 extremely important*). The model comprised ten dimensions (e.g., positive reputation in lesbian, gay, bisexual, transgender [LGBT] community, services are available anonymously, clinic is not LGBT-specific; see Table 2).

### Statistical analysis

To answer our research question about differences by concealment of same-sex behavior, we stratified descriptive statistics for socio-demographics, testing behaviors, sexual behaviors, risk perceptions, and the sexual health service model by whether participants had disclosed same-sex behavior to an HCP at least once in their lives when seeking advice for sexual health issues (disclosure vs. non-disclosure). For our first aim to assess factors associated with disclosure vs. non-disclosure, we performed bivariate analyses using T tests and chi-square tests to evaluate the difference between the two groups on socio-demographic and sexual health related behavior. Significant variables were then analyzed in multivariate logistic regression (odds ratios, 95% confidence intervals) to evaluate independent factors associated with disclosing same-sex behavior to HCPs. Variables with *p*-values < .10 in bivariate analyses were selected for inclusion in an initial logistic regression model and odds ratios with *p*-values < .05 were included in the final multivariate model. We calculated mean scores for each dimension and across all 10 dimensions of sexual health service and likewise stratified by disclosure for bivariate analyses. All data analyses were completed using IBM SPSS Statistics 20 (IBM, Armonk, NY, USA).

## Results

Of 362 respondents, 1.4% (*n* = 5) of respondents had missing data. Of the 357 respondents in the final sample, 68.1% (*n* = 243) had disclosed same-sex behavior to an HCP at least once in their lives when seeking advice for sexual health issues (Table 1).

**Table 1:** Socio-demographic and behavioral characteristics of Chinese MSM, stratified by history of disclosure of same-sex behavior to HCP when seeking sexual health services (*N* = 357)

	Ever disclosed *n* (%) 243 (68.1)	Never disclosed *n* (%) 114 (31.9)	*p*	Overall *(N = 357)*
Socio-demographics				
Age (*M*, *SD*)	30.6, 9.2	33.4, 9.6	0.009	31.5, 9.4
City currently living in				
Shanghai	33 (82.5)	7 (17.5)	< 0.001	40
Beijing	79 (77.5)	23 (22.5)		102
Changsha	47 (74.6)	16 (25.4)		63
Guangzhou	84 (55.3)	68 (44.7)		152
Local residency permit	71 (64.5)	39 (35.5)	0.390	110
College education or above	163 (68.5)	75 (31.5)	0.811	238
Full-time employment (20 missing data)	158 (65.0)	85 (35.0)	0.505	243
Household				
I live by myself	75 (60.0)	50 (40.0)	0.009	125
I live with a partner	56 (64.4)	31 (35.6)		87
I live with roommate(s) or relatives	74 (81.3)	17 (18.7)		91
I do not have a stable home/Other	36 (66.7)	18 (33.3)		54
Married	40 (66.7)	20 (33.3)	0.879	60
Average monthly income (USD)				
Low and middle income (≤442)	41 (74.5)	14 (25.5)	0.350	55
Middle income (443–1474)	94 (69.6)	41 (30.4)		135
High income (≥1474)	108 (64.7)	59 (35.3)		167
Sexuality				
Sexual attraction				
More to women than to men/Only to women	7 (58.3)	5 (41.7)	0.732	12
To women and men equally	27 (62.8)	16 (37.2)		43
More to men than to women	108 (69.7)	47 (30.3)		155
Only to men	101 (68.7)	46 (31.3)		147
Sexual identity				
Bisexual	74 (66.7)	37 (33.3)	0.896	111
Gay	166 (68.6)	76 (31.4)		242
Heterosexual/Other	3 (75.0)	1 (25.0)		4
Current intimate sexual relationship with a woman	38 (61.3)	24 (38.7)	0.292	62
Testing behaviors				
Frequency of HIV testing				
Never	18 (42.9)	24 (57.1)	0.001	42
Once per year or less	70 (65.4)	37 (34.6)		107
About 2–3 times per year	80 (73.4)	29 (26.6)		109
At least 4 times per year	75 (75.8)	24 (24.2)		99
Frequency of STI testing				
Never	63 (60.6)	41 (39.4)	0.028	104
Once per year or less	90 (65.2)	48 (34.8)		138
About 2–3 times per year	47 (75.8)	15 (24.2)		62
At least 4 times per year	43 (81.1)	10 (18.9)		53
Sexual behavior in past 6 months				
Anal sex positioning (15 missing data)				
Any receptive	154 (73.3)	56 (26.7)	0.009	210
Insertive only	78 (59.1)	54 (40.9)		132
Sex with men or women or both				
Only with men	172 (69.4)	76 (30.6)	0.664	248
More with men than with women	36 (63.2)	21 (36.8)		57
With women and men equally	9 (56.2)	7 (43.8)		16
More with women than with men/Only with women	8 (66.7)	4 (33.3)		12
Haven’t had sex	33 (78.6)	9 (21.4)		24
Number of male sex partners				
0–1	102 (66.7)	51 (33.3)	0.140	153
2–5	118 (66.7)	59 (33.3)		177
≥ 6	23 (85.2)	4 (14.8)		27
HIV-positive male sex partner (15 missing data)				
None	116 (66.7)	58 (33.3)	0.393	174
I don’t know	105 (69.5)	46 (30.5)		151
Yes, partner on ART	8 (80.0)	2 (20.0)		10
Yes, partner not on ART	3 (42.9)	4 (57.1)		7
Condomless anal sex	106 (72.6)	40 (27.4)	0.128	146
Commercial sex with male sex partners	18 (66.7)	9 (33.3)	1.000	27
STI symptoms /diagnosis	17 (73.9)	6 (26.1)	0.647	23
Recreational drug use	3 (75.0)	1 (25.0)	1.000	4
Group sex with male sex partners	27 (71.1)	11 (28.9)	0.716	38
Perceptions of HIV and risk				
HIV in social networks	92 (66.7)	46 (33.3)	0.812	138
Likelihood of HIV acquisition (M, SD) ^2^	2.1, 0.7	2.2, 0.7	0.157	2.1, 0.7
Worry about HIV acquisition (M, SD) ^3^	3.1, 0.9	2.8, 0.9	0.003	3.0, 0.9

### Socio-demographics

As seen in Table 1, participants ranged from 18 to 85 years old (*M* = 31.5, *SD* = 9.4). Overall, two-thirds of participants self-identified as gay and about a third as bisexual. Just over two-thirds reported a college degree or higher. Most were not married, and most were employed full-time. Nearly half reported high-income.

### Sexual health related behavior

About one in ten had never tested for HIV and nearly one in three had never tested for an STI. More than half reported receptive anal sex in the past 6 months, and many were unaware of the HIV status of their partner(s). On average, participants thought they were unlikely to become infected with HIV in the next five years and endorsed worry about acquiring HIV. About two-fifths knew someone who had acquired HIV in the last two years.

**Fig. 1:**
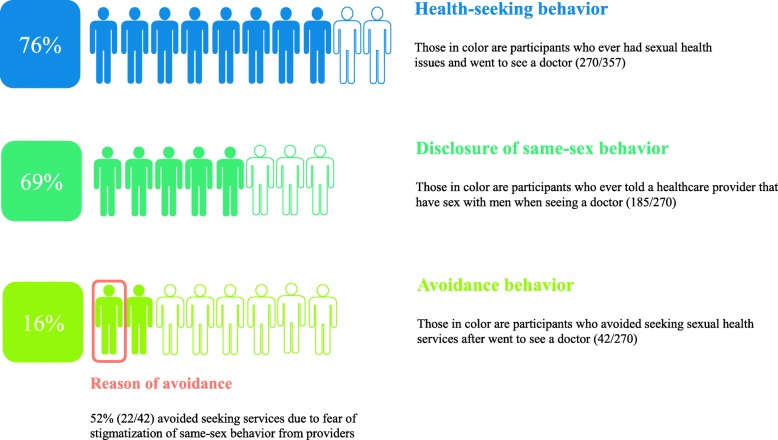
MSM’s sexual health related behavior (*N *= 357). The flow displays MSM’s sexual health issues, health-seeking behavior, and disclosure of same-sex behavior

Three quarters reported seeing an HCP for a sexual health issues that had interfered with sex. Of these, most had disclosed same-sex behavior to an HCP at least once in their lives while seeking sexual health services. Among those who avoided seeking care for such an issue, half cited the main barrier to be fear of stigma toward same-sex behavior by HCPs (Fig. 1).

### Demographic differences by disclosure of same-sex behavior to HCPs

Those who had ever sought help for sexual health and disclosed same-sex behavior to their HCPs were significantly more likely, at *p* < .10, to report younger age, living with other people, not having a local residency permit, more frequent HIV testing and STI testing, greater worry about HIV acquisition, more receptive anal penetration, and differences by city of residence as compared to those MSM who had never disclosed (Table 1). We did not find evidence that disclosure of same-sex behavior to HCPs was associated with any other sociodemographic variables.

### Factors independently associated with disclosure of same-sex behavior to HCPs

In a multivariate model of factors significant in bivariate analyses, MSM who were older (*aOR* = .96; *95% CI*: .93–.99) and those who reported insertive sex only (*aOR* = .47; *95% CI*: .28–.78) had significantly lower odds of having disclosed same-sex behavior to their HCPs. Those who stated that they were afraid of contracting HIV (*aOR* = 1.39; *95% CI*: 1.05–1.84) had higher odds of having disclosed (Table 2).

**Table 2:** Factors associated with disclosure of same-sex behavior to HCP (*N* = 357)

	Crude OR (*95% CI*)	*P*	Adjusted OR (*95% CI*) ^*^	*P*
Socio-demographics				
Age (in years)	0.97 (0.95–0.99)	0.010	0.96 (0.93–0.99)	0.005
City currently living in				
Shanghai	1			
Beijing	0.73 (0.29–1.86)	0.508		
Changsha	0.62 (0.23–1.68)	0.351		
Guangzhou	0.26 (0.11–0.63)	0.003		
Household				
I live by myself	1		1	
I live with a partner	1.20 (0.68–2.12)	0.520	1.00 (0.52–1.90)	0.991
I live with roommate(s) or relatives	2.09 (1.54–5.49)	0.001	1.79 (0.84–3.79)	0.130
I do not have a stable home/Other	1.50 (0.75–2.99)	0.249	1.18 (0.54–2.61)	0.678
Testing behaviors				
Frequency of HIV testing				
Never	1		1	
Once per year or less	2.52 (1.22–5.23)	0.013	2.16 (0.85–5.49)	0.108
About 2–3 times per year (every 4–6 months)	3.68 (1.75–7.74)	0.001	2.67 (0.99–7.22)	0.053
At least 4 times per year (every 3 months)	4.17 (1.94–8.95)	<0.001	1.82 (0.62–5.35)	0.275
Frequency of STI testing				
Never	1		1	
Once per year or less	1.22 (0.72–2.07)	0.459	1.23 (0.60–2.51)	0.572
About 2–3 times per year (every 4–6 months)	2.04 (1.01–4.11)	0.047	1.13 (0.44–2.89)	0.801
At least 4 times per year (every 3 months)	2.80 (1.27–6.18)	0.011	2.77 (0.90–8.51)	0.075
Sex risk in past 6 months				
Sex role				
Any receptive “0” or “0.5”	1		1	
Insertive “1” only	0.53 (0.33–0.83)	0.006	0.47 (0.28–0.78)	0.004
Worry about HIV acquisition (vs. not worried)	1.43 (1.13–1.81)	0.003	1.39 (1.05–1.84)	0.020

*Adjusted for location in the multivariate model

### Sexual health services MSM value by disclosure of same-sex behavior to HCPs

**Fig. 2:**
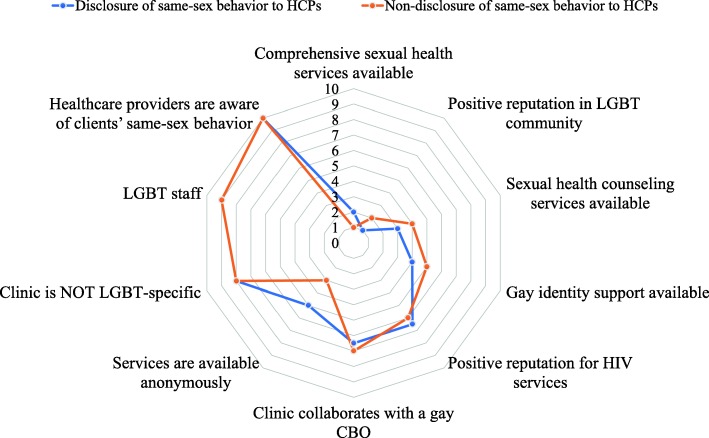
Dimensions of sexual healthcare services that gay men value grouped by disclosure of same-sex behavior to HCP (*N* = 357). The spider map presents sexual health services that MSM valued when seeing HCPs

The overall average importance score for all dimensions of the “ideal” sexual health clinic was *M* = 3.73 (*SD* = .23) and ranged from 3.34 to 4.01 on the 5-point Likert scale (Table 3). *Ever disclosed* respondents had higher scores than *never disclosed* respondents, with no significant mean differences for the overall model (*M* = 3.80 vs. *M* = 3.60, *p* = .07). However, the two groups differed significantly across three dimensions (in order of magnitude): *sexual health counseling services available* (*M* = 3.99 vs. *M* = 3.65, *p* = .002), *gay identity support available* (*M* = 3.91 vs. *M* = 3.62, *p* = .016), and *clinic collaborates with a gay CBO* (*M* = 3.81 vs. *M* = 3.56, *p* = .036). Both groups valued *positive reputation in the LGBT community* and *comprehensive sexual health services available* as the top two services. The largest gap in ranking, albeit not statistically significant by mean differences, was *services are available anonymously*, ranked 5th in the disclosure group versus 3rd in the non-disclosure group (*M* = 3.88 vs. *M* = 3.67, *p* = .081). *Healthcare providers are aware of clients’ same-sex behavior* and *LGBT staff* ranked as the bottom two services in importance for both groups (Fig. 2).

**Table 3:** Sexual healthcare delivery model stratified by history of disclosure of same-sex behavior to HCP (*N* = 357)

	Ever disclosed *n* (%) 243 (68.1)		Never disclosed *n* (%) 114 (31.9)			Overall (*N* = 357)	
	Importance score^*^ (*M, SD*)	Ranking (1–10)	Importance score (*M, SD*)	Ranking (1–10)	t-test score (*p)*	Importance score (*M, SD*)	Ranking (1–10)
Positive reputation in LGBT community	4.06, 1.00	1	3.89, 0.95	2	−1.49 (0.138)	4.01, 0.99	2
Comprehensive sexual health services available	4.05, 0.92	2	3.95, 0.98	1	−1.00 (0.319)	4.02, 0.94	1
Sexual health counseling services available	3.99, 0.91	3	3.65, 1.08	4	−3.12 (0.002)	3.88, 0.98	3
Gay identity support available	3.91, 1.04	4	3.62, 1.05	5	−2.42 (0.016)	3.82, 1.05	4
Services are available anonymously	3.88, 1.02	5	3.67, 1.13	3	−1.75 (0.081)	3.81, 1.06	5
Clinic collaborates with a gay CBO	3.81, 0.99	6.5	3.56, 1.09	7	−2.11 (0.036)	3.73, 1.03	7
Positive reputation for HIV services	3.81, 1.03	6.5	3.61, 1.16	6	−1.72 (0.086)	3.75, 1.08	6
Clinic is NOT LGBT-specific	3.63, 1.05	8	3.47, 1.00	8	−1.37 (0.172)	3.58, 1.03	8
LGBT staff	3.45, 1.13	9	3.35, 1.03	9	−0.82 (0.415)	3.42, 1.10	9
Healthcare providers are aware of clients’ same-sex behavior	3.40, 1.16	10	3.22, 1.03	10	−1.45 (0.149)	3.34, 1.12	10

*1 = Not at all important, 5 = Extremely important

## Discussion

We examined whether disclosure of same-sex behavior to HCPs was associated with demographic and sexual health factors among MSM who visited gay-oriented CBOs for HIV VCT services in four cities in China, and relatedly, whether their past disclosure while seeking sexual health services was associated with their preferences for hypothetical sexual health services. Overall in our sample, MSM reported a high prevalence of sexual health issues, elevated worry about becoming infected with HIV, and extraordinarily high proportions of seroconversions within their social networks. In multivariate logistic regression, MSM who reported younger age and greater worry about HIV acquisition were more likely to have disclosed their same-sex behavior to HCPs. Past disclosure was also associated with slightly greater interest in three out of ten dimensions for sexual health services.

### Disclosure of same-sex behavior to HCPs and testing and sexual behaviors of MSM

A large majority (68.1%) of respondents reported that they had previously experienced a sexual health issue that interfered with their sex life, sought care, and disclosed their same-sex behavior to an HCP, highlighting the importance of understanding the sexual health of sexual-minority populations beyond the narrow lens of HIV. This high prevalence of disclosure is close to those reported in a recent systematic review [2] with a median of 61% across 15 studies. However, this review was largely conducted in high-income countries. Compared to a 2014 nationwide online study [11] in China, in which only 16% MSM reported disclosure of their sexual orientation to HCPs, our prevalence of disclosure is much higher. We attribute the difference to recruitment within gay-oriented CBOs, an indication that gay-specific services may be associated with greater disclosure. However, while most men who experience a sexual health issue sought care and most disclosed their same sex behavior to providers, half of those who did not seek healthcare cited anticipated stigma by HCPs as the primary reason for not doing so, suggesting continued need for interventions that build the capacity of HCPs to deliver MSM-competent care. This finding is generally in line with the findings from a 2017 study that three-fifths of MSM who saw a physician in the last two years in China have ever experienced healthcare discrimination [10].

Though all participants were recruited from MSM-focused VCT clinics, a sizable minority (11.8%) reported never having tested for HIV. In China, MSM-focused CBOs may function as a place for social support related to sexual health, such as counselling related to sexual orientation or obsessive fear about HIV acquisition, rather than solely HIV testing. These participants may also have visited the VCT clinic for the first time, and remained pre-contemplative or contemplative about HIV testing itself. Further research could better describe this important subpopulation, to better understand the connection between never testing for HIV and visiting MSM-focused CBOs.

### Ideal sexual health clinic for MSM

Those men who ever disclosed same-sex behavior placed a higher value on sexual health counseling services, support for gay identity, and gay CBO oriented clinic than those never who had never disclosed. In the context of previous studies of disclosure and HIV/STI services among MSM [11, 14, 15, 20] our sexual health service model highlighted the importance of cultural and psychological supports in sexual health services. Disclosure of same-sex behavior by MSM to HCPs is integral to appropriate sexual health services, (e.g. HPV vaccination) and likely depends on provider-level interactions [21, 22].

The availability of comprehensive sexual health services was the most valued dimension in our sample. A “one-stop shopping” approach, clustering a variety of tailored sexual health services for MSM in the same venue [23, 24], have yet to be provided in the Chinese context [10, 25]. It will be critical to identify models that integrate sexual health services with HIV prevention with the upcoming introduction of HIV pre-exposure prophylaxis (PrEP) into China for key populations such as MSM [26]. However, there are structural barriers to achieving this. One example is that primary care physicians, whose services are underused since most people see specialists first, may be more competent in managing health services or facilitating referrals for MSM clients [10]. Other barriers stem from STI specialists. Though WHO has recommended provider-initiated HIV testing and counseling since 2007 [27], and HIV testing rates in STI clinics have steadily increased [28], low perception of the prevalence of disease is still common among STI care providers [13]. These STI specialists may experience difficulties in recognizing their MSM clients’ needs and providing competent services.

The largest gap in the service dimensions related to anonymity. Not surprisingly, those who had not previously disclosed same-sex behavior prized service dimensions that connote less risk of disclosure. Compared to MSM who had disclosed, they reported slightly lower preferences for sexual health counseling, gay identity support, reputation for HIV services, collaboration with a gay CBO, and awareness among HCPs of clients’ same sex behavior. While anonymity distinguished the two groups, HCPs being aware of clients’ same-sex behavior (ranked 10th in both groups) was the least valued service for all. Substantial discrimination and stigma toward same-sex behavior in healthcare settings could be a key reason why most MSM prefer to avoid disclosing same-sex behavior when going to HCPs for sexual health issues [10]. A few studies have shown that ignorance of LGBT lifestyles and sexual practices, lack of appropriate language and probing, preconception of MSM clients’ sexual orientation, and homophobic attitudes from HCPs are key barriers to disclosure [2, 25]. Structural intervention and training for HCPs can be effective strategies to facilitate disclosure of same-sex behaviors in MSM, which could ultimately resolve the gaps between the demand for sexual health services and the delivery of sexual health services [2, 3].

Our study has several limitations. First, we investigated disclosure of same-sex behavior and sexual health service model based on client-side perceptions and did not incorporate HCPs’ perspectives. Second, recall bias may limit how participants answered questions related to health-seeking experiences. Third, the participants were recruited from an HIV VCT clinic, which might introduce selection bias, as CBOs in these four cities in China include MSM-friendly staff. Fourth, this was a cross-sectional study and no causal relationships can be inferred.

## Conclusions

Our hypothesis that MSM who had disclosed versus never disclosed same-sex behavior would differ in the value they placed on different dimensions of sexual health services was partially borne out. A cross-sectional survey to elicit opinions and preferences on service delivery dimensions of more hidden MSM populations, who likely have much lower rates of disclosure to HCP, could be conducted through online recruitment methods and bring more nuance to our understanding of diversity within MSM populations in China. As health authorities in China decide on implementation models for PrEP delivery and, specifically how to integrate PrEP into existing health services, attention should be paid to the preferences of target populations in order to develop comprehensive, patient-centric and LGBT-friendly services. Well-designed pragmatic trials to compare service delivery models of PrEP could be helpful to inform introduction and scale up of PrEP so that this powerful biomedical intervention can be delivered in a way that maximizes its potential to rapidly achieve epidemiological impact.

## Data Availability

The datasets used and/or analysed during the current study are available from the corresponding author on reasonable request.

## Abbreviations

CBO: Community-based Organization
GPP: Good Participatory Practices
HCPs: Healthcare Providers
HIV: Human Immunodeficiency Virus
LGBT: Lesbian, Gay, Bisexual, Transgender
MSM: Men Who Have Sex with Men
PrEP: Pre-exposure Prophylaxis
STIs: Sexually Transmitted Infections
VCT: Voluntary Counselling and Testing
